# Atrial fibrillation after pulmonary lobectomy for lung cancer affects long-term survival in a prospective single-center study

**DOI:** 10.1186/1749-8090-7-4

**Published:** 2012-01-10

**Authors:** Andrea Imperatori, Giovanni Mariscalco, Giuditta Riganti, Nicola Rotolo, Valentina Conti, Lorenzo Dominioni

**Affiliations:** 1 Department of Surgical and Morphological Sciences, Center for Thoracic Surgery, Varese University Hospital, University of Insubria, Varese, Italy; 2 Department of Surgical and Morphological Sciences, Cardiac Surgery Unit, Varese University Hospital, University of Insubria, Varese, Italy

**Keywords:** Pulmonary lobectomy, Lung cancer, Atrial fibrillation, Arrhythmia, Prognosis, Mortality

## Abstract

**Background:**

Atrial fibrillation (AF) after thoracic surgery is a continuing source of morbidity and mortality. The effect of postoperative AF on long-term survival however has not been studied. Our aim was to evaluate the impact of AF on early outcome and on survival > 5 years after pulmonary lobectomy for lung cancer.

**Methods:**

From 1996 to June 2009, 454 consecutive patients undergoing lobectomy for lung cancer were enrolled and followed-up until death or study end (October 2010). Patients with postoperative AF were identified; AF was investigated with reference to its predictors and to short- and long-term survival (> 5 years).

**Results:**

Hospital mortality accounted for 7 patients (1.5%), while postoperative AF occurred in 45 (9.9%). Independent AF predictors were: preoperative paroxysmal AF (odds ratio [OR] 5.91; 95%CI 2.07 to 16.88), postoperative blood transfusion (OR 3.61; 95%CI 1.67 to 7.82) and postoperative fibro-bronchoscopy (OR 3.39; 95%CI 1.48 to 7.79). Patients with AF experienced higher hospital mortality (6.7% vs. 1.0%, p = 0.024), longer hospitalization (15.3 ± 10.1 vs. 12.2 ± 5.2 days, p = 0.001) and higher intensive care unit admission rate (13.3% vs. 3.9%, p = 0.015). The median follow-up was 36 months (maximum: 179 months). Among the 445 discharged subjects with complete follow-up, postoperative AF was not an independent predictor of mortality; however, among the 151 5-year survivors, postoperative AF independently predicted poorer long-term survival (HR 3.75; 95%CI 1.44 to 9.08).

**Conclusion:**

AF after pulmonary lobectomy for lung cancer, in addition to causing higher hospital morbidity and mortality, predicts poorer long-term outcome in 5-year survivors.

## Background

Atrial fibrillation (AF) remains the most common medical complication after thoracic surgery, with an incidence ranging from 10% to 20% after pulmonary lobectomy, and as much as 40% after pneumonectomy [1-7]. Postoperative AF has been shown to predict worse prognosis, being correlated with higher hospital morbidity and mortality and with a considerable increase of hospital stay and cost [1-6]. However, the prognostic implications of this arrhythmia after pulmonary lobectomy for lung cancer remain controversial. None of the studies examining the consequences of postoperative AF has managed to present compelling data supporting an independent association between this arrhythmia and late mortality, because postoperative survival was examined only up to 36 months [4,5].

The primary aim of this study was to assess the impact of AF on early outcome and on survival > 5 years from pulmonary lobectomy for lung cancer. Factors associated with AF development after lobectomy were also investigated.

## Materials and methods

### Population and study design

Between January 1996 and June 2009, 473 consecutive patients undergoing lobectomy for primary lung cancer at Varese University Hospital were considered for this study; none of the operations was an emergency. Of these patients 19 were excluded from analysis because they had chronic AF (n = 8), pace-maker devices (n = 4), or incomplete data (n = 7). Patients with a history of paroxysmal AF, but in sinus rhythm at operation, were included [3]. The final study cohort comprised 454 patients (81.3% male), with mean age of 65.4 ± 8.8 years (range 28 to 84). Patient characteristics are listed in Table 1.

**Table 1:** Patient characteristics

**Predictor**^**a**^	All patients (n = 454)	Patients without AF (n = 409)	Patients with AF (n = 45)	*p V*alue
*Demographic*				
Mean age, year	65.4 ± 8.8	65.0 ± 8.9	68.6 ± 6.8	0.008
Male, *n (%)*	369 (81.3)	332 (81.2)	37 (82.2)	0.864
BMI, kg/m^2^	25.6 ± 4.1	25.7 ± 4.2	24.7 ± 3.3	0.116
*Comorbidities*				
Paroxysmal AF, *n (%)*	45 (9.9)	38 (8.8)	7 (15.5)	0.182
CAD, *n (%)*	56 (12.3)	45 (11.0)	11 (24.4)	0.009
Prior AMI, *n (%)*	15 (3.3)	12 (2.9)	3 (6.7)	0.178
Hypertension, *n (%)*	183 (40.3)	161 (39.4)	22 (48.9)	0.216
Diabetes, *n (%)*	58 (12.8)	53 (13.0)	5 (11.1)	0.999
Dyslipidemia, *n (%)*	71 (15.6)	60 (14.7)	11 (24.4)	0.087
Current smokers, *n (%)*	182 (40.1)	164 (40.1)	18 (40.0)	0.990
PVD, *n (%)*	115 (25.3)	106 (25.9)	9 (20.0)	0.386
CVA, *n (%)*	19 (4.2)	16 (3.9)	3 (6.7)	0.421
*Baseline biochemical data *				
Creatinine, mg/dL	1.0 ± 0.5	1.0 ± 0.4	1.2 ± 1.1	0.287
Hb, g/dL	13.8 ± 1.6	13.8 ± 1.5	13.6 ± 1.8	0.352
*Baseline respiratory data *				
FEV_1_, % of predicted	88.1 ± 21.9	88.2 ± 21.7	86.9 ± 24.1	0.716
PaO_2_, mmHg	94.5 ± 21.9	94.3 ± 20.9	96.3 ± 29.2	0.558
*Preoperative therapy*				
β-blockers, *n (%)*	36 (7.9)	29 (7.1)	7 (15.6)	0.046
Calcium antagonists, *n (%)*	58 (12.8)	51 (12.5)	7 (15.6)	0.556
ACE-Inhibitors, *n (%)*	67 (14.8)	59 (14.4)	8 (17.8)	0.547
ARBs, *n (%)*	33 (7.3)	29 (7.1)	4 (8.9)	0.659
Statins, *n (%)*	45 (9.9)	39 (9.5)	6 (13.3)	0.429
Neoadjuvant chemotherapy, *n (%)*	30 (6.6)	28 (6.8)	2 (4.4)	0.756

^a ^For continuous variables, mean ± SD (standard deviation); for categorical variables, number (percent)

*ACE *angiotensin converting enzyme, *AF *atrial fibrillation, *AMI *acute myocardial infarction, *ARB *angiotensin receptor blocker, *BMI *body mass index, *CAD *coronary artery disease, *CVA *cerebrovascular accident, *FEV*_1 _forced expiratory volume in 1 second, *Hb *haemoglobin, *PaO*_2 _partial arterial oxygen pressure, *PVD *peripheral vascular disease

Throughout the study period the patients' data were prospectively recorded in a computerized database. The latter included information about demographics, comorbidities, medical and surgical history, preoperative respiratory and cardiac testing, operative details and postoperative events during the hospital stay (Table 1 and 2). After discharge, follow-up was conducted according to the American College of Chest Physicians (ACCP) guidelines [8], with physical examination and imaging study (chest x-rays (CXR) or computed tomography (CT)) every 6 months for 2 years and then annually. For patients who died during follow-up, the date of death was recorded. For patients lost to follow-up, the vital status were ascertained at the end of study, by linkage with the Lombardy Region Health System Registry. The vital status of residents outside this region were ascertained by contacting family members or the respective general practitioner. Survival follow-up was closed on October 30, 2010.

**Table 2:** Peri- and post-operative data

**Predictor**^**a**^	Patients without AF (n = 409)	Patients with AF (n = 45)	*p V*alue
*Lung cancer characteristics *			
Cancer location, *n (%)*			0.011
Right lung	236 (57.7)	17 (37.8)	
Left lung	173 (42.3)	28 (62.2)	
UICC Stage, *n (%)*^b^			0.568
Stage I	211 (54.0)	24 (54.5)	
Stage II	86 (22.0)	7 (15.9)	
Stage III/IV	94 (24.0)	13 (29.5)	
Histology, *n (%)*			0.281
Squamous cell ca	140 (34.2)	21 (46.7)	
Adenocarcinoma	209 (51.1)	21 (46.7)	
Large cell	24 (5.9)	1 (2.2)	
Other	36 (8.8)	2 (4.4)	
*Perioperative data*			
PaO_2_, mmHg	85.3 ± 30.8	86.9 ± 16.3	0.729
Ventilation time, h	3.2 ± 0.7	3.2 ± 0.7	0.540
Inotropes, *n (%)*	23 (5.6)	2 (4.4)	0.999
Blood transfusions, *n (%)*	36 (8.8)	13 (28.9)	< 0.001
*Postoperative data*			
Reoperation for bleeding, *n (%)*	3 (0.7)	0 (0)	0.999
FBS, *n (%)*	31 (7.1)	13 (24.4)	< 0.001
AMI, *n (%)*	2 (0.5)	2 (4.4)	0.051
CVA, *n (%)*	7 (1.7)	2 (4.4)	0.221
AKI, *n (%)*	14 (3.4)	3 (6.7)	0.232
Respiratory failure, *n (%)*	3 (0.7)	5 (11.1)	< 0.001
Pneumonia, *n (%)*	5 (1.2)	2 (4.4)	0.146
Length of stay, days	12.2 ± 5.2	15.3 ± 10.1	0.001
ICU admission n (%)	16 (3.9)	6 (13.3)	0.015
Hospital Mortality n (%)	4 (1.0)	3 (6.7)	0.024

^a ^For continuous variables, mean ± SD (standard deviation); for categorical variables, number (percent)

^b ^UICC stage [11] not available for the 19 (4.2%) patients with tumor type other than NSCLC

*AKI *acute kidney injury (according RIFLE criteria), *AMI *acute myocardial infarction, *CVA *cerebrovascular accident (stroke + transient ischemic attack), *FBS *fibrobronchoscopy, *ICU *intensive care unit, *PaO*_2 _partial arterial oxygen pressure, *UICC *Union International Contre le Cancer

With the aim to analyze mortality profile of all discharged patients, we recorded the cause of death by linkage with the Varese Province Mortality Registry. The cause of death was classified by disease groups, according to the International Classification of Diseases, Edition IX (ICD-IX) as follows: cardiovascular diseases (ICD-IX: 390-459); lung cancer (ICD-IX: 162.2-162.9); all cancers other than lung cancer (ICD-IX:140-162.0, 163-239); all other causes of deaths.

The protocol of this study was in compliance with the local Institutional Review Board and received full approval. Written informed consent was obtained from the participants of this study.

### Patient management

All patients underwent preoperative clinical cardiologic and anesthesiologic evaluation, CXR and CT, and pulmonary function tests. Preoperative medications, including β-blockers, diuretics, antihypertensives, statins, and calcium-channel blockers were routinely omitted on the day of the operation and were restarted on postoperative day one, unless clinically contraindicated. Operability was determined according to established guidelines for lobectomy [9,10]. All pulmonary resections were performed by open thoracotomy, by the same thoracic surgical team throughout the study period. Standardized surgical approach and anesthesiologic management were used and remained constant during the study. Briefly, short-term antibiotic prophylaxis was routinely administered intravenously (ampicillin/sulbactam 3 gr) before anaesthesia. An epidural catheter for postoperative pain relief was offered to all patients and premedication with midazolam was done before induction of general anaesthesia. After administration of rocuronium bromide (0.15 mg/kg) and orotracheal intubation with double-lumen tube, anaesthesia was maintained by 50% O_2 _and 2% sevoflurane. Mediastinal sampling lymphadenectomy was routinely performed. Pathological lung cancer staging was assessed according to the 1997 TNM classification [11]. Two chest tubes were placed on water seal at the end of the operation and removed when no air leaks were present and pleural drainage output was < 150 mL/24 h. Postoperative pain control was achieved mainly by epidural analgesia and/or by systemic opioids combined with non-steroidal anti-inflammatory drugs. Low molecular weight heparin was administered for 2-4 weeks postoperatively.

After surgery patients were transferred to a general intensive care unit (ICU) for the first 12/24 hours. Heart rate, electrocardiography (ECG), central venous and arterial pressures, and acid-base blood gases were continuously monitored during the ICU stay. Inotropic support was provided if the ventricular contractility was markedly impaired. Perioperative need of blood products was determined on an individual, patient-by-patient basis; in general, blood transfusions were administered when haemoglobin was < 8 g/dL. Postoperative fibrobronchoscopy (FBS) was performed in case of lung atelectasis, and in order to obtain bronchial secretion samples for microbiological examinations. All patients had an active program of postoperative physiotherapy including deep-breathing exercises.

### AF monitoring and definition

Cardiac rhythm assessment followed the daily practice of an integrated clinic encompassing ICU and ward level, sharing the same routines and data collection system. Patients were monitored by continuous ECG during a minimum of 48 hours postoperatively. Subsequent monitoring was by repeated daily observations by nurses and physicians, at least every 2 hours. In case of rhythm disturbance reported by nurse or patient, a 12-lead ECG recording was obtained, and continuous ECG monitoring was restarted if necessary. Additional recordings were collected at clinical suspicion of AF. The arrhythmia was defined by physician assessment, on the basis of a telemetry strip or a 12-lead ECG recording. Amiodarone, either orally or intravenously administered, constituted the standard pharmacological treatment of AF. Digoxin was administered if necessary to reduce high ventricular rate. In case of AF recurrence, the same protocol was applied. In this study the definition of postoperative AF includes the successfully treated AF as well as AF persistent at discharge.

### Statistical analysis

Extracted database variables were tabulated using Microsoft Excel^® ^(Microsoft Corp, Redmond, WA) and statistical analysis was computed using SPSS, release 16.0 for Windows^® ^(SPSS Inc., Chicago, IL). Continuous variables were tested for normal distribution by the Kolmogorov-Smirnov test and compared between groups with unpaired Student's *t *test for normally distributed values; otherwise, the Mann-Whitney *U *test was employed. In case of dichotomous variables, group differences were examined by chi-square or Fisher exact tests as appropriate.

A stepwise logistic regression model was developed to identify variables predicting postoperative AF. The model was built with univariable predictors of AF with *p *value ≤ 0.15. The stepwise approach was confirmed by backward and forward methods. The strength of the association of variables with the dependent one was estimated by calculating the odds ratio (OR) and 95% confidence intervals (CI). The model was calibrated by the Hosmer-Lemeshow goodness-of-fit test, while model discrimination was evaluated by using the area under the receiver operating characteristic (ROC) curve.

Kaplan-Meier estimates and log-rank test were performed for the postoperative mortality rate comparison of patients with or without postoperative AF. Mortality hazard ratios (HRs) were generated by a multivariable Cox regression analysis, using univariable Cox predictors with p < 0.15 (Table 3). Patients who died within 30 days of operation were excluded from the final analysis of survival.

**Table 3:** Univariable Cox Predictors of postoperative mortality

Variable	OR	95%CI	*p *Value
*Demographic*			
Age, years	1.02	1.01 - 1.03	0.042
Female	0.68	0.48 - 0.95	0.025
BMI, kg/m^2^	0.98	0.94 - 1.02	0.256
*Cardiac data*			
Paroxysmal AF	1.74	1.01 - 2.98	0.044
CAD	1.76	0.93 - 3.31	0.080
Prior AMI	1.41	0.98 - 2.01	0.061
*Comorbidities*			
Hypertension	1.12	0.87 - 1.43	0.387
Diabetes	1.73	1.23 - 2.41	0.001
Dyslipidemia	1.21	0.83 - 1.76	0.326
COPD	0.99	0.77 - 1.27	0.931
Current smokers	1.27	0.99 - 1.64	0.064
PVD	1.03	0.77 - 1.37	0.836
Preop CVA	1.41	0.79 - 2.51	0.249
CRF	1.17	0.29 - 4.70	0.826
*Other data*			
Creatinine (basal), mg/dL	1.20	0.89 - 1.62	0.216
Hb, g/dL	0.95	0.88 - 1.03	0.210
FEV_1 _< 80%	0.74	0.58 - 0.96	0.025
*Cancer characteristics*			
Right Lobectomy	1.16	0.91 - 1.48	0.235
Neoadjuvant chemotherapy	1.04	0.62 - 1.75	0.884
Stage UICC^a^	1.96	1.52 - 2.53	< 0.001
Histology^b^	0.91	0.71 - 1.16	0.452
*Postoperative Data*			
Inotropes	1.79	0.98 - 3.29	0.060
Blood transfusion	1.51	1.02 - 2.22	0.037
FBS	1.11	0.69 - 1.78	0.658
Postop AMI	1.98	0.28 - 14.18	0.493
Postop CVA	3.26	1.45 - 7.35	0.004
AKI	1.17	0.62 - 2.20	0.632
POPAF	1.41	0.95 - 2.09	0.087

^a ^Classes I and II vs. classes III and IV (class I and II as a reference group)

^b ^Adenocarcinoma vs. others carcinoma (others as a reference group)

*AKI *acute kidney injury, *AF *atrial fibrillation, *AMI *acute myocardial infarction, *BMI *body mass index, *CAD *coronary artery disease, *COPD *chronic obstructive pulmonary disease, *CRF *chronic renal failure, *CVA *cerebrovascular accident, *FBS *fibrobronchoscopy, *FEV*_1 _forced expiratory volume in 1 second, *Hb *hemoglobin, *POPAF *postoperative atrial fibrillation, *PVD *peripheral vascular disease, *UICC *Union International Contre le Cancer.

A *p *value < 0.05 was considered statistically significant. Results are expressed as mean ± standard deviation (SD) for continuous variables and frequencies for the categorical ones.

## Results

### AF and early outcome

Hospital mortality accounted for 7 (1.5%) subjects, while AF occurred in 9.9% of patients (45 of 454) and its frequency peaked on the second postoperative day (69% of cases). Mean AF duration was 9.2 ± 7.1 hours (range: 1-24). Of the 45 patients with postoperative AF, 29 (64%) had a single arrhythmia episode, while 16 (35%) experienced multiple episodes. Among the 45 patients with postoperative AF, 27 (60%) were treated by amiodarone, 7 (16%) by digoxin, 4 (9%) by calcium-channel blocker, and 2 (4%) by beta-blocker. The remained 5 patients were treated with a simple correction of the electrolyte imbalance (potassium). In all cases the choice of medication was based on Cardiologist prescription. At discharge, persistent AF was present in 2 of 45 (4.4%) patients.

Patients with AF compared to patients without it were older and more frequently had a history of paroxysmal AF or coronary artery disease (p = 0.008, p < 0.001 and p = 0.009, respectively) (Table 1). Other comorbidities, pulmonary function tests and preoperative medications, with the exception of β-blocker agents, did not reveal significant differences between patients with AF and without it. No correlation was observed between postoperative AF and neo-adjuvant chemotherapy, lung cancer stage and histological type of cancer (p = 0.756, p = 0.568, p = 0.281 respectively). Subjects with cancer location in the left lung were more frequently affected by AF (p = 0.011). Postoperatively, patients with AF had a higher prevalence of respiratory failure (p < 0.001), of postoperative FBS (p < 0.001) and required more frequently blood transfusions (p < 0.001). Moreover, patients with AF had longer hospital stay (p = 0.001), higher ICU admission rate (p = 0.015), and higher hospital mortality (p = 0.024) (Table 2).

At multivariate analysis, independent predictors of postoperative AF were preoperative paroxysmal AF (OR 5.91, 95%CI 2.07 to 16.88), need of peri-operative blood transfusion (OR 3.61, 95%CI 1.67 to 7.82), and postoperative FBS (OR 3.39; 95%CI 1.48 to 7.79). The Hosmer-Lemeshow goodness-of-fit test (χ^2 ^[1 d.f.] = 0.61, p = 0.433) and ROC analysis (AUC of 0.70) revealed good calibration and discrimination for the multivariate analysis.

### AF and late survival

**Figure 1:**
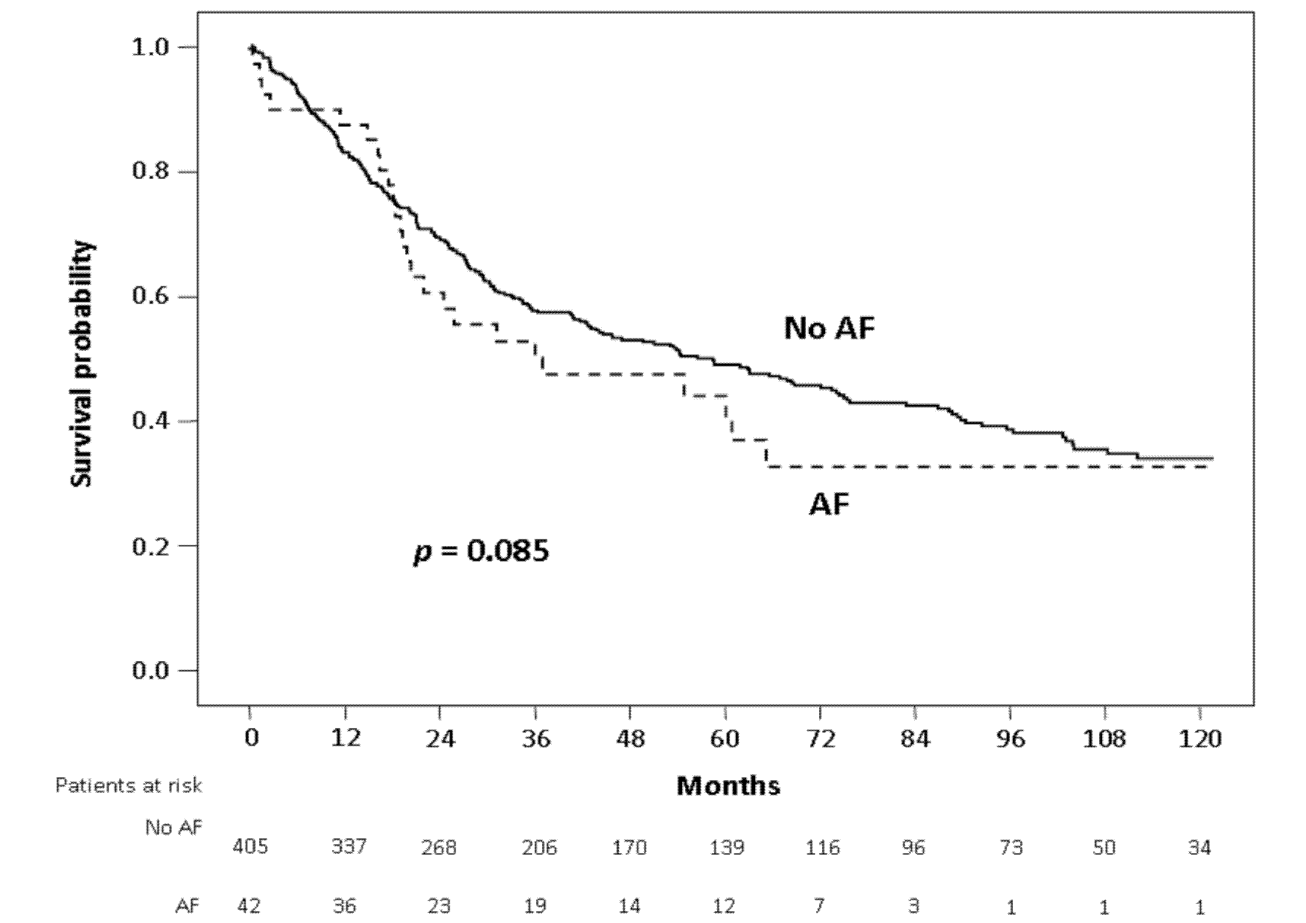
**Kaplan-Meier survival curves of patients with atrial fibrillation (AF) and without AF (No AF)**.

Follow-up was completed for 445/447 (99.6%) patients discharged after resection, with median follow-up of 36 months (maximum: 179 months). Kaplan-Meier analysis of subjects without AF revealed 1-, 5- and 10-year overall survival of 99%, 49% and 34%, respectively, similar to 98%, 42%, and 31% survival of patients with AF (p = 0.085) (Figure 1).

At multivariable Cox regression analysis of 445 discharged patients with complete follow-up, postoperative AF was not an independent predictor of late mortality (HR 1.17; 95%CI 0.76 to 1.79), while independent predictors were: lung cancer UICC stage (HR 2.09; 95%CI 1.60 to 2.74), diabetes (HR 1.64; 95%CI 1.17 to 2.29), male gender (HR 1.51; 95%CI 1.07 to 2.13), and age (HR 1.02; 95%CI 1.00 to 1.04) (Table 4). Among the 151 patients alive at 5 years from cancer resection, the multivariable Cox regression analysis revealed that postoperative AF was the strongest independent predictor (HR 3.75; 95%CI 1.44 to 9.81) along with forced expiratory volume in 1 second (FEV_1_) < 80% of predicted (HR 2.07; 95%CI 1.09 to 3.93) (Table 4).

**Table 4:** Independent predictors of postoperative mortality

Predictor	*p *Value	HR	95% CI
*In 445 discharged patients*			
UICC cancer stage^a^	< 0.001	2.09	1.60 - 2.74
Diabetes	0.004	1.64	1.17 - 2.29
Male gender	0.018	1.51	1.07 - 2.13
Age^b^	0.021	1.02	1.00 - 1.04
*In 151 5-year survivors*			
Postoperative AF	0.007	3.75	1.44 - 9.81
FEV_1 _< 80%	0.027	2.07	1.09 - 3.93

^a ^Classes I and II vs. classes III and IV (class I and II as a reference group)

^b ^By one-year increment

*CI *confidence interval, *HR *hazard ratio, *FEV*_1 _forced expiratory volume in 1 second, *UICC *Union Internationale Contre le Cancer

Among the 445 discharged subjects with complete follow-up, death from cardiovascular diseases occurred more frequently in patients affected by postoperative AF than in those without it (21% vs.10%, p = 0.073). Among 151 5-year survivors, a similar difference of cardiovascular disease mortality was also observed (40% *vs*. 21%, *p *= 0.336).

## Discussion

Despite improvements in surgical and anesthesiological techniques, the incidence of AF after thoracic surgery has remained substantially unchanged over the past two decades [1-7]. Although several studies have analysed the risk factors for this arrhythmia and possible preventive strategies, its exact pathophysiology has not been elucidated yet. Few data are available regarding the impact of AF on survival after thoracic surgery [4-7]. Our study confirms the negative impact of AF on hospital mortality after lobectomy for lung cancer; in addition, it provides the first evidence that patients with postoperative AF who survive 5 years have a significantly reduced long-term survival.

Our data also identified preoperative paroxysmal AF, postoperative FBS and blood transfusions as independent predictors of postoperative AF. While paroxysmal AF and transfusion requirement are well-known AF risk factors, because of the electrical and histological abnormalities of patient atrial tissue and because of the amplified inflammatory response associated with transfusion of blood components [2,12-14], the correlation between postoperative FBS and AF has not been reported previously. A possible explanation of such correlation is the peri-operative stress of the FBS procedure, resulting in a hyperadrenergic state with increased levels of catecholamines. The latter enhance triggered activity and automaticity, which are key factors in the development of atrial arrhythmia [15,16].

An intriguing observation of the present study was an increased AF occurrence in patients undergoing left lobectomy compared with those subjected to right one (62% vs. 38%). A plausible reason could be related to the increased manipulation and increased trauma of the left cardiac structures (left atrial auricular and left pulmonary vein) [17]. However, this statistical relationship was not confirmed at multivariable level.

Our data confirm that AF following lobectomy for lung cancer increases early postoperative mortality and causes significant adverse effects, prolonging the length of ICU and hospital stay [2-4]. Postoperative AF was here associated with three- to six-fold increased risk of both hospital mortality and ICU admission, and with two- to three-day increase in total hospital length.

A relevant finding of our study was the negative impact of postoperative AF on long-term survival. Previous investigations on the subject focused on the peri-operative period and failed to include the analysis of long-term survival, because postoperative follow-up was interrupted after about 3 years [4,5]. The association between postoperative AF and postoperative survival is controversial [4-6]. Amar and co-workers [5] first demonstrated that early supraventricular tachydysrhythmias (SVT) were associated with reduced postoperative survival, in a population of 78 patients with non-small cell lung carcinoma. At the conclusion of that study (median follow-up: 17 months), only 1 of 10 patients with SVT was alive, whereas 39 of 68 (57%) who did not develop SVT were alive (p = 0.01) [5]. Murthy and colleagues [6] reported the association of postoperative AF with increased risk of late adverse outcomes in 198 patients after esophagectomy. In that case series, drawn from 921 patients, median survival was shorter for those affected by AF compared with controls (11.5 vs. 14.5 months); however, when hospital mortality was excluded from analysis, survival was not different (14.5 vs. 16.9 months) [6]. Cardinale and colleagues [4], after 233 lung cancer operations with a mean follow-up of 18 ± 8 months, recorded no difference of 3-year mortality between patients with and without AF.

All the above mentioned studies analysing a possible direct association between postoperative AF and mortality after thoracic surgery, however, have limitations due to heterogeneous cancer populations, small sample sizes, incomplete matching or exclusion of many patients from analysis, and follow-up not extended beyond three years [4-6]. Our study had much longer follow-up (median 36; maximum 179 months). Among 5-year survivors we found that postoperative AF was an independent predictor of poor long-term survival.

The mechanisms by which postoperative AF may cause mortality in later years are difficult to analyze. Despite attempts to account for confounding mechanisms, it is possible that AF is associated with mortality because it usually occurs in patients with a more severe comorbidity profile [1-3]. Plausible mechanisms supporting a direct effect of postoperative AF include heart failure and the potential AF recurrence with attendant thromboembolic sequelae [18,19].

Our findings are consistent with the data presented by Groth and co-workers [20]. The risk of dying for lung cancer exceeds the risk of dying of cardiovascular disease immediately after lung surgery, but this relation diminished with time. Being postoperative AF a mirror of cardiac status, an important implications for NSCLC survivors is the need for a long-term surveillance and prophylaxis of arrhythmias along with the planned lung follow-up.

There are limitations to the present study. Firstly, this is a single center study and its design is retrospective, although the data were prospectively collected. Secondly, the statistical analysis is limited by the large difference between the number of patients with AF and of those without it. Thirdly, the association we observed between AF and late mortality does not necessarily indicate causation, although studies on the general population affected by chronic AF and studies reporting the outcome of cardiac surgery patients with postoperative AF, revealed a direct AF effect in causing late mortality [18,21]. In our study subgroup analysis of AF patients by tumor stage, and by cause of death was not feasible, due to the small number of subjects in each subgroup. Similarly, we cannot evaluate the possible role of systematic mediastinal lymph node dissection, because we routinely performed mediastinal sampling lymphadenectomy. Finally, we did not collect information about post-discharge AF recurrence, nor did we control for the effect of drug administration after patient discharge, due to unreliable information on anti-arrhythmic medications and long-term use of anticoagulation therapy. Because of these limitations, the proposed mechanisms explaining the statistically significant association that we found between postoperative AF and poorer 5-year survival remain speculative. Despite these limitations, to date our study is the largest capturing the late deleterious effects of AF and examining the clinically relevant question of whether AF after pulmonary lobectomy for cancer is associated with increased long-term mortality.

## Conclusion

Postoperative AF predicts poorer long-term survival in 5-year survivors after pulmonary lobectomy for cancer, in addition to causing higher hospital morbidity and mortality. After pulmonary lobectomy for cancer, a long-term surveillance and prophylaxis of arrhythmia seem justified.

## List of abbreviations

ACCP: American College of Chest Physicians; ACE: angiotensin converting enzyme; AF: atrial fibrillation; AKI: acute kidney injury; AMI: acute myocardial infarction; ARB: angiotensin receptor blocker; BMI: body mass index; CI: confidence intervals; CAD: coronary artery disease; COPD: chronic obstructive pulmonary disease; CT: computed tomography; CRF: chronic renal failure; CVA: cerebrovascular accident; CXR: chest x-rays; ECG: electrocardiography; FEV_1_: forced expiratory volume in 1 second; FBS: fibrobronchoscopy; Hb: haemoglobin; HR: hazard ratio; ICD: International Classification of Diseases; ICU: intensive care unit; NSCLC: non small cell lung cancer; OR: odds ratio; PaO_2_: partial arterial oxygen pressure; POPAF: postoperative atrial fibrillation; PVD: peripheral vascular disease; ROC: receiver operating characteristic; SD: standard deviation; SVT: supraventricular tachydysrhythmias; UICC: Union International Contre le Cancer.

## Competing interests

The authors declare that they have no competing interests.

## Authors' contributions

AI and LD designed the study, analyzed and interpreted the data and wrote the manuscript; GM performed the statistical analysis, analyzed the data and wrote the manuscript; GR contributed to the design the study and acquisition of data; NR and VC helped to draft the final manuscript and added important comments to the paper.

All authors read and approved the final manuscript.
